# New UV-source catalogs, UV spectral database, UV variables and science tools from the GALEX surveys

**DOI:** 10.1007/s10509-018-3277-2

**Published:** 2018-02-28

**Authors:** Luciana Bianchi, Alexander de la Vega, Bernard Shiao, Ralph Bohlin

**Affiliations:** 1 Department of Physics and Astronomy, The Johns Hopkins University, Baltimore, MD 21210 USA; 2 Space Telescope Science Institute, Baltimore, USA

**Keywords:** Astronomical data bases: surveys, Astronomical data bases: catalogs, Stars: white dwarfs, Galaxy: stellar content, Stars: variables, Stars: novae, cataclysmic variables, Stars: oscillations (including pulsations), Ultraviolet: stars

## Abstract

We present a new, expanded and improved catalog of Ultraviolet (UV) sources from the GALEX All-Sky Imaging survey: GUVcat_AIS (Bianchi et al. in Astrophys. J. Suppl. Ser. 230:24, 2017). The catalog includes 83 million unique sources (duplicate measurements and rim artifacts are removed) measured in far-UV and near-UV. With respect to previous versions (Bianchi et al. in Mon. Not. R. Astron. Soc. 411:2770 2011a, Adv. Space Res. 53:900–991, 2014), GUVcat_AIS covers a slightly larger area, 24,790 square degrees, and includes critical corrections and improvements, as well as new tags, in particular to identify sources in the footprint of extended objects, where pipeline source detection may fail and custom-photometry may be necessary. The UV unique-source catalog facilitates studies of density of sources, and matching of the UV samples with databases at other wavelengths.

We also present first results from two ongoing projects, addressing respectively UV variability searches on time scales from seconds to years by mining the GALEX photon archive, and the construction of a database of ∼120,000 GALEX UV spectra (range ∼1300–3000 Å), including quality and calibration assessment and classification of the grism, hence serendipitous, spectral sources.

## Introduction

The Galaxy Evolution Explorer (GALEX, Martin et al. 2005; Morrissey et al. 2007) has surveyed the sky at ultraviolet (UV) wavelengths for almost a decade (2003–2013). It performed nested surveys with different area coverage and depth (Bianchi 2009, 2011, 2014; Bianchi et al. 2014, 2017), yielding a database of 582,968,330 measurements^1^ in far-UV (FUV, $\lambda _{\mathit{eff}}\sim 1528~{\mathring{\mathrm{A}}}$, 1344–1786 Å) and near-UV (NUV, $\lambda _{\mathit{eff}}\sim 2310~{\mathring{\mathrm{A}}}$, 1771–2831 Å), from 100,865 “visits” in imaging mode.^2^ As of now, no major UV *survey* is planned in the near future, therefore the GALEX database remains to date not only unprecedented, but the only extensive resource for studies of hot stars, extragalactic objects such as star-forming galaxies and low-redshift QSOs, as well for planning future observations and estimating the yield from future instruments (e.g., Bianchi 2016).

For this reason, Bianchi et al. (2017) have produced an improved, enhanced version of the GALEX catalog of UV sources, from the imaging surveys. We will briefly recall the relevant improvements implemented in GUVcat in Sect. 2, and outline the science applications.

GALEX has also collected 125,564 grism UV spectra (range ∼1300–3000 Å, resolution ∼200 (FUV)–118 (NUV)) of 117,195 distinct sources,^3^ a homogeneous template tenfold larger than the IUE target sample and extending it to ∼2 dex fainter fluxes, with only slightly lower resolution, albeit with highly varying quality owing to the serendipitous nature of the grism data. The grism mode offers the advantage of obtaining at once spectra of all sources in the ∼1.2 degree diameter field that are bright enough to be detected, without *a priori* selection of individual targets, hence a unique potential for novel discoveries. Yet, this unique resource has hardly been exploited, due to the poor calibration and lack of robust quality assessment of the complex grism spectral extraction, and the difficulty of identifying and classifying sources for a large serendipitous sample. We describe in Sect. 3 our ongoing effort to produce a science-ready database and related tools to facilitate science applications of the GALEX spectra, including our own first science objective which is to derive extinction curves in all sight-lines sampled by these data.

Third and most relevant in a current astrophysics context, is the potential to mine the 130TB archive of individual photons recorded by GALEX’s MCP detectors. Until recently, only integrated images and photometry from each observation were available in the public archive. It is now possible to search through the GALEX recorded photon list, and extract photometry with chosen time integrations for a given source. Such facility opens up unprecedented opportunities for investigations of short-term UV variability, serendipitous and unbiased, over samples orders-of-magnitude larger than any dedicated variability search could afford, and the only one in the UV. We have undertaken two large projects to search for variability on all time scales afforded by the database, from seconds to the >9 years of GALEX operations, but especially focussing on the fast cadences which remain largely unexplored. In Sect. 4 we present results from the early part of the project, the necessary identification and mitigation of instrumentally-induced (“artifact”) variability, which is conspicuous and previously unreported because no such short-time-scale investigations have been undertaken on a major scale during the mission operations phase. We also show examples of previously undetected fast variations in two different types of hot stellar sources.

## New GALEX catalogs of UV sources

The GALEX field of view is ${\approx}1.2^{\circ }$ diameter ($1.28/1.24^{\circ }$, FUV/NUV), and the spatial resolution is ${\approx} 4.2/5.3^{\prime \prime }$ (Morrissey et al. 2007). In imaging mode, GALEX has performed sky surveys with different depth and coverage in FUV and NUV simultaneously, until the FUV detector stopped working in May 2009 (Bianchi 2009, see Bianchi et al. 2014, 2017 for sky coverage and source distributions of the surveys).

As mentioned in the previous section, the GALEX database includes almost 600 million source measurements from individual observations (or “visits”), these were combined into over 200 million measurements from “coadded” visits, which still include repeated measurements of a number of sources. For matching UV-source samples with other catalogs, or for examining source densities, a unique-source catalog is needed. Bianchi et al. (2017) discovered problems in a number of the original database “coadds”, therefore we rebuilt a new catalog of unique-sources using a combination of coadds and visit-level data. This new version, $\mathit{GUVcat}$, supersedes the previous versions of Bianchi et al. (2011a, 2014) and should be used instead. Another improvement is the addition of two tags to flag sources that fall in the footprint of extended objects such as large galaxies or Galactic stellar clusters: in many cases the GALEX pipeline fails to resolve point-like sources over the extended galaxies disks or in the crowded cluster cores, and severe mismatches result between FUV and NUV photometry of the same observation, in addition to missing most sources in these areas; the effect is very severe as can be seen in Figures 5a–d of Bianchi et al. (2017): GUVcat flags $\mathit{INLARGEOBJ}$ and $\mathit{LARGEOBJSIZE}$ allow to easily exclude these areas.

Because the reference paper (Bianchi et al. 2017) and the catalog have appeared at the time of submitting this work, we will not repeat details here. GUVcat_AIS, with related science tools, is accessible from LB’s web site http://dolomiti.pha.jhu.edu/uvsky, from MAST Casjobs, and soon from from CDS’s Vizier.

The UV unique-source catalog facilitates studies of density of sources, and matching of the UV samples with databases at other wavelengths: SDSS and Pan-STARRS which provide five optical bands each, enabling classification of Galactic and extragalactic sources from UV-to-optical colors (Bianchi et al. 2011a, 2011b, 2018a, 2018b, 2018c, 2018d); the first Gaia source and Gaia TGAS releases, available to date only for the brightest sources, add precise position and G-band photometry for bright sources, and direct distance measurements for the very bright ones (Bianchi et al. 2018a, 2018b, 2018d).

## The GALEX spectroscopic database

**Fig. 1:**
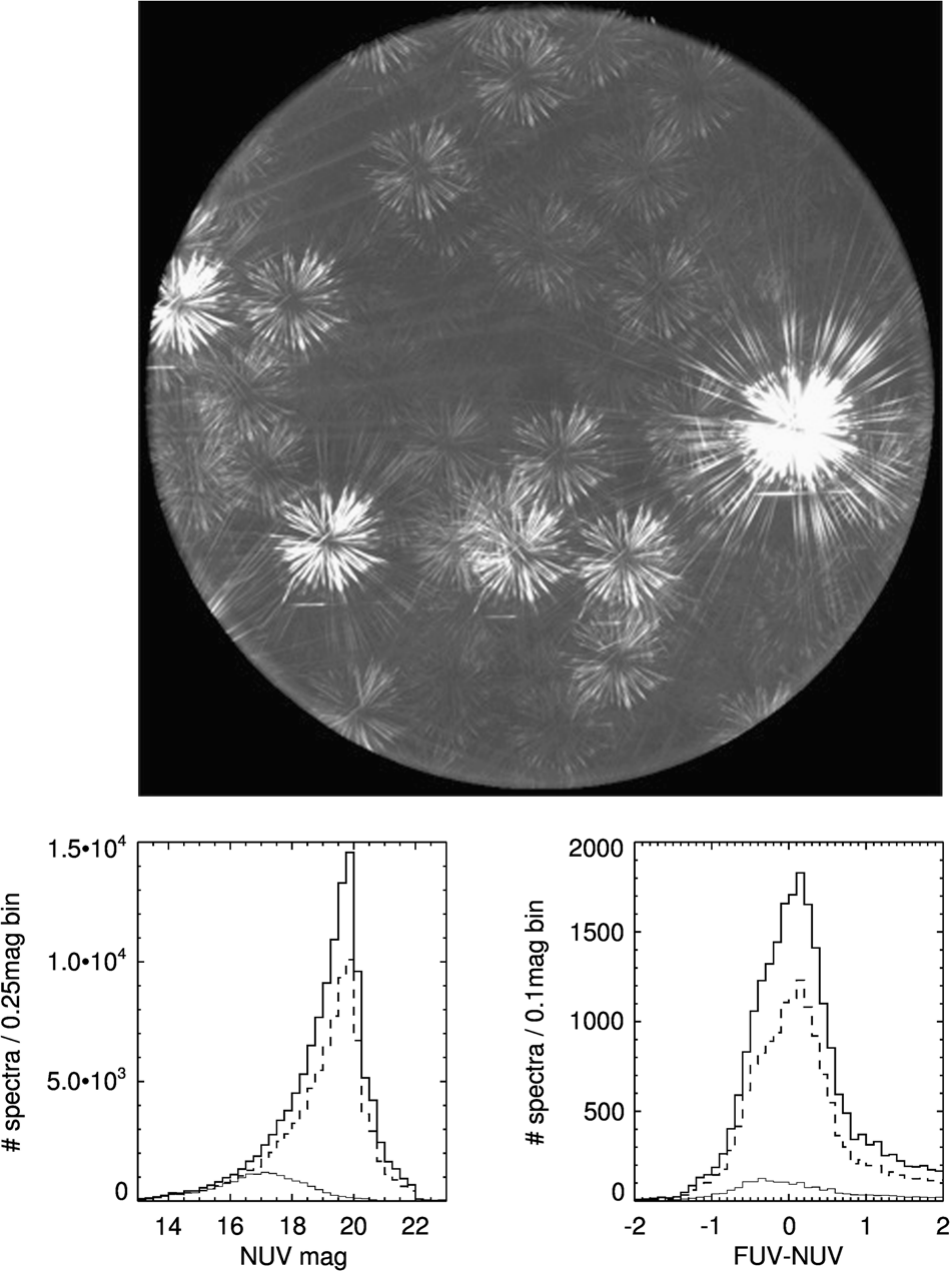
Top: A typical GALEX grism image coadded from exposures taken at several grism orientations. In some special cases orientations were optimized to reduce spectral overlap (e.g., Bianchi et al. 2012). Bottom: Distribution of UV magnitude and FUV–NUV color for sources with GALEX spectra. The solid thick line is the total sample, the dashed histogram is the point-like sample, and the thin line the sources with spectra having S/N per pixel >5. A limit of GALEX NUV=22 ABmag corresponds to a flux $\mathrm{F}_{\lambda } = 3.5\times 10^{-17}~\mbox{ergs}\,\mbox{sec}^{-1}\,\mbox{cm}^{-2}\,{\mathring{\mathrm{A}}}^{-1}$. For comparison, the faintest objects observed with IUE, to our knowledge, have fluxes from a few up to $10 \times 10^{-15}~\mbox{ergs}\,\mbox{sec}^{-1}\,\mbox{cm}^{-2}\,{\mathring{\mathrm{A}}}^{-1}$: these were the most luminous stars in nearby galaxies, and ∼18 hours exposure in each IUE camera (corresponding roughly to the GALEX FUV and NUV ranges) were needed to detect a spectrum (Bianchi et al. 1991)

GALEX also had a spectroscopic observing mode, in which a grism inserted in the optical path produced FUV and NUV slitless spectra of all sources in the field. A direct image was also taken for each spectroscopic observation: source positions from the reference image are used to extract grism spectra and associate them with source coordinates, as well as to assess overlap in portions of spectra from nearby sources and exclude these portions from the final combined spectra. To minimize losses from spectral overlap, each spectroscopic observation was taken with several grism orientations, and the extracted good (non-overlapping) spectral portions combined (Fig. 1). The spectra have a resolution of about 118 (NUV) and 200 (FUV), and a total useful range from ∼1340 to 2830 Å, with a cross over from FUV to NUV at ∼1820 Å. The 1D extracted spectra are recorded in a range 1300–2997.5 Å with a 2.5 Å sampling.

The homogeneous set of >100,000 GALEX UV spectra distributed in fields all over the sky can enable several investigations; our own goal is the derivation of UV extinction curves for ${\gtrsim} 10^{3}$ sight-lines throughout the Milky Way (MW), yielding information on properties of interstellar dust, in particular the small grains component, that cannot be obtained at other wavelengths, and, furthermore, validating extinction maps derived from vastly larger photometric samples.

**Fig. 2:**
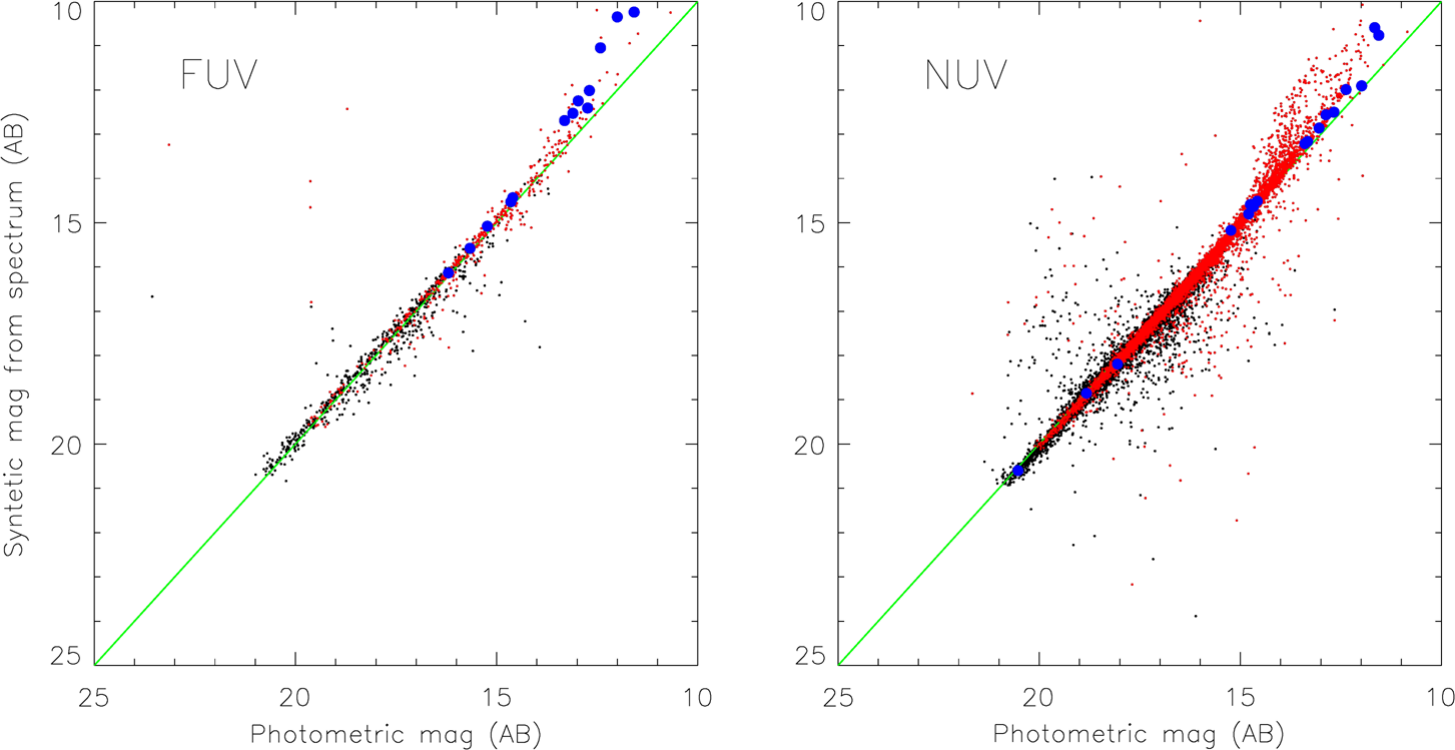
Comparison of GALEX magnitudes derived from the GALEX spectra and measured in the corresponding direct images. Black and red dots indicate spectra with median S/N larger than 10 and 5 respectively. The large blue dots are CALSPEC comparison stars (note that some are in the non-linear regime of GALEX brightness). The green line marks the $1:1$ correspondence
- Although the majority of spectral sources have only one spectrum in the GALEX database, over 6,000 sources have two spectra, over 400 have three, and 11 sources have four repeated spectra. These repeated spectra allow an “internal” comparison to be performed for evaluating repeatability, while the comparison with the STIS and IUE data mentioned above provide an assessment of the flux calibration scale. Examples of repeated spectra are shown in Fig. 3. The result is that repeatability does not simply scale with signal, as is usually the case in slit spectrographs such as HST spectrographs and IUE; we are examining a number of additional parameters to establish correlations, and therefore additional criteria, applicable to the entire dataset.

To make this study, and others, possible, we must identify (if a known optical counterpart exists) and classify all sources in the grism, hence serendipitous, GALEX spectroscopic sample, and assess quality and flux calibration for each spectrum. A critical step for this objective and a result of general utility is the verification of extraction stability and flux calibration of GALEX spectra. Given the complexity of the grism extraction, standard parameters such as signal-to-noise (S/N) are not always sufficient as quality criteria. We have undertaken therefore a comprehensive assessment with many-fold approaches, briefly outlined below:

- comparisons by cross-strapping hot white dwarfs of intermediate brightness between GALEX and HST UV calibration standards (CALSPEC calibration database): the CALSPEC spectra are “gold reference” standards, and can be used to reconcile the calibration scales among different instruments (e.g., Bohlin and Bianchi 2018, and references therein). There are eleven HST (and six IUE) CALSPEC standards useful for comparison, however in many cases their flux falls in the GALEX saturation or non-linearity regime. This comparison is of no use for parametrizing the stability of GALEX spectral extraction but it is essential to bring the GALEX flux calibration scale on the HST standard flux scale.
- An additional, larger sample is obtained by matching the GALEX spectral sources against the IUE database. A first caveat is that IUE coordinates do not have a homogeneous precision: using a very inclusive (large) match radius can produce spurious matches, while using a restrictive radius may cause to miss real matches. We have found a total number of about 2,900 IUE datasets matching about 130 GALEX spectral sources; less than half of them are useful. The IUE faint limit of flux is close to GALEX brightness limit for detector safety or to the non-linearity regime (Fig. 1—bottom panel). Nonetheless, this overlapping sample is sufficient to bring (together with the CALSPEC sample mentioned above) the GALEX flux calibration on a consistent scale with other datasets, so that the two collections of IUE and GALEX spectra, covering a brighter and fainter sample respectively, with similar wavelength range, resolution and quality, will eventually represent a benchmark collection of UV templates.
- For a comparison which can be extended to all GALEX spectra we have computed FUV and NUV synthetic magnitudes convolving the GALEX spectra with the transmission curves of GALEX direct imaging, and compared them with the photometric measurements in the corresponding direct images. A reference direct image is taken for each grism-mode image, and used to define the position of the spectral extraction strips, as well as to exclude portions where spectra from nearby sources overlap. The result of the comparison is shown in Fig. 2, where only the spectra with median signal-to-noise >5 (black dots) and >10 (red dots) and ellipticity <0.3 are shown^4^: 1123 FUV and 16556 NUV spectra. Unuseable portions of spectra are not accounted for in the comparison shown in Fig. 2, this factor is relevant for spectra with lower signal-to-noise. In the figure the CALSPEC calibration standards are marked as large blue dots: note that in the brightest-fluxes regime there is a deviation from the $1:1$ correspondence between photometry and spectral measurements: such deviation characterizes the response non-linearity at high countrates.

**Fig. 3:**
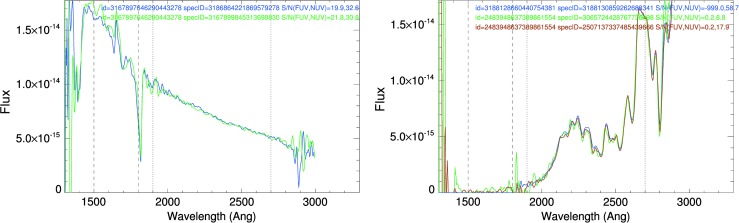
Examples of GALEX spectra of a hot (left, PG 1300-099) and a cool (right, GSC2.2 S0332231180) star, with repeated spectra. Vertical lines indicate approximate ranges (dashed for FUV, dotted for NUV) where the spectra are most free of artifacts and of most reliable quality: data within such limits are used to estimate science tags in the spectra master catalog, such as e.g., maximum flux in various ranges (Bianchi et al. 2018b). The consistency is good for several cases, as the ones shown here, but other cases show discrepancies much larger than the countrate-based S/N

Results from all these assessments (still partly in progress) will be included in a comprehensive paper, in which we will also release the resulting entire database of quality-assessed, recalibrated spectra with source identification, classification, and multi-wavelength corollary data (SDSS, PanSTARSS, 2MASS, Gaia, …) when available (Bianchi et al. 2018b).

## UV time-domain science

GALEX images available in the MAST archive are the result of pipeline reconstruction by integrating each photon event recorded over the length of the observation. Most variability studies so far have been based on comparing measurements between different observations (e.g., Conti et al. 2014 and references therein), with a few exceptions (e.g., Welsh et al. 2006, 2007, 2011; Browne et al. 2009; Wheatley et al. 2012; Browne et al. 2013). The exposure times of GALEX individual observations (“visits”) range from about 100 secs up to ∼30 minutes, the duration of an “eclipse” (i.e., the useful observing time) during each orbit. Several sources have repeated observations, making it possible to explore long-term variability on timeframes up to the duration of the GALEX mission (e.g., Conti et al. 2014).

**Fig. 4:**
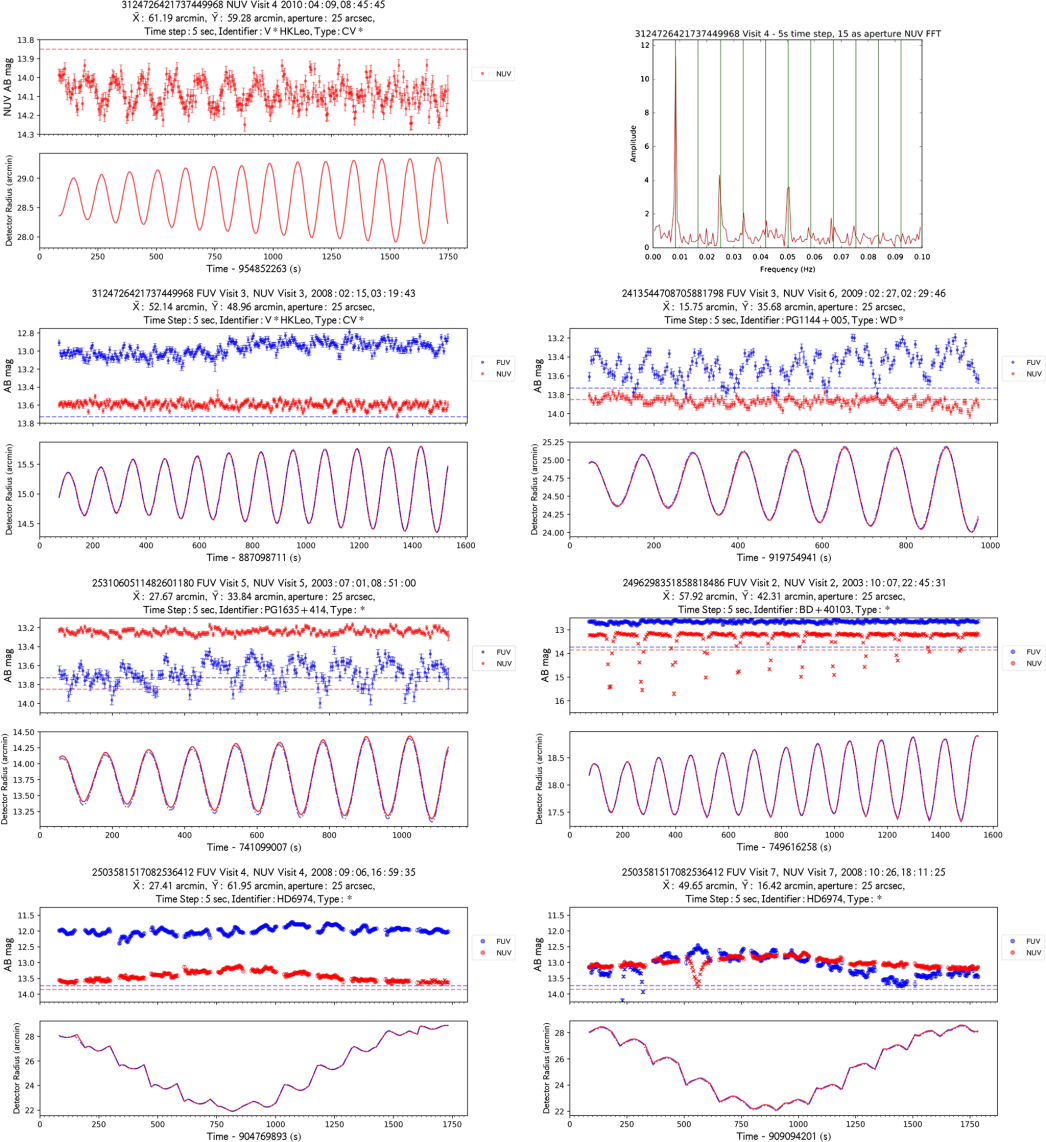
Examples of false variability produced by instrumental effects in GALEX light curves. From top-left: The light curve of GALEX ID=3124726421737449968 with time integrations of 5 seconds in the top plot, and the dithering motion shown as distance from the field center (“Detector Radius”) in the lower plot; to the right, the FT plot of the light curve, where vertical green lines mark the frequency of the dithering position variation and its harmonic frequencies. In the second row, a light curve of the same source from a different observation (left), which displays also a “jump” i.e. a one-time sudden change in flux level in FUV, and another example (right) where the quasi-sinusoidal variation induced by the dithering pattern is more conspicuous in FUV. Another example follow where dithering-induced instability affects more the FUV flux (third-row left), the countrate in either channel being in the saturated regime and in the ∼linear regime respectively in the last two examples. In the third row—right, an example of sharp, fast drops in brigthness. In the last row, two examples of flux “sagging” during the visit, again related to the dithering motion. As is the case for other artifacts, one or both channels may be affected, in the same way or in different ways. NUV data are plotted in red, FUV in blue; crosses mark data-points affected by hot spots, horizontal dashed lines mark the countrates above which non-linearity sets in. In the x-axis, “Time” in seconds is “GALEX timestamp”, = t_UNIX-315964800, where t_UNIX = elapsed seconds since January 1, 1970

**Fig. 5:**
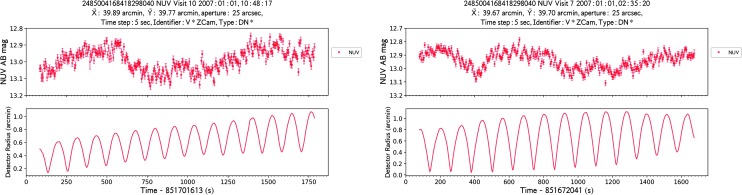
Two GALEX NUV observations of Z Cam, taken about 8 hours apart on 2007 Jan. 1, displaying variations by about 0.3 mag on timescales of a few hundred seconds, and possibly faster, more subtle flickering. With the light curves (top plots), we also show the spacecraft dithering motion (lower plots), showing that the source variations are unrelated to the dithering pattern, although such motion contributes some additional variability (left), which limits the characterization of possible instabilities on time scales close to the dithering frequency. Colors and symbols as in Fig. 4

GALEX used photon-counting microchannel plate detectors to record photon events with a timing precision of five-thousandths of a second. It has recently become possible to mine the entire photon archive, and to integrate selected portions of GALEX images with a specified time “bin” thanks to the $gPhoton$ package (Million et al. 2016), effectively producing light curves across the duration of each GALEX observation (Figs. 4–5). This database opens entirely new possibilities for time-domain science in the UV, complementing dedicated facilities at optical wavelengths. We have undertaken essentially two large projects, aimed on the one hand at improving in various ways the $\textit{gPhoton}$ capabilities (Million et al. 2018), and to construct large collections of UV light curves for different types of variables objects, notably late-type flaring stars (e.g., Million et al. 2017), hot variables such as pulsators, binaries, etc. (Bianchi et al. 2018c, Tucker et al. 2017).

In order to prepare and vet the methodology for database-wide comprehensive projects, we tested our analysis procedures on a sample of 350 bright, hot stellar sources, with a total of over 6,000 observations; we chose very bright sources so that we can explore the shortest time scales, with good S/N even for few-seconds integrations; the sample, with limiting magnitude of 14 in FUV and NUV, spans the saturation/non-linear- and the linear-response regime, therefore we can test the limitations from high-countrate non-linearity as well. We discovered a number of previously unreported, both spatial and temporal instrumental instabilities, which [occasionally] induce various types of characteristic artificial variability in the light curves. We performed a thorough investigation to characterize these artifacts (de la Vega et al. 2018), in order both to identify and flag such events in automated screening of large light-curve samples (several thousand at a time), establish the causes or the conditions in which such artifacts do and do not occur, and devise algorithms to extricate physical variations of the sources from instrumentally-induced variations.

We refer for details to de la Vega et al. (2018), who performed a characterization of the light curves based on Fourier-Transform (FT) analysis and various period searches, and searches for correlations with instrumental parameters; we only summarize here the major types of artifacts affecting the GALEX time-domain investigations. Below we will show also examples of real variability. The main types of artifacts detected by de la Vega et al. (2018), are:

- Strictly periodic, triangle-shape or quasi-sinusoidal variations with periods of ∼120 sec and amplitudes up to a few tenths of a magnitude (Fig. 4, top panels). They may occur in either or both detectors (during simultaneous FUV, NUV observations), but most frequently in NUV; they last through the entire observation in most cases but may also be limited to one portion of the observation. They are caused by the dithering motion of the spacecraft during the exposure (a spiral pattern of amplitude of the order of 1arcmin), used to minimize effects of pxl-to-pxl variations, hot spots and other defects. This correlation was revealed by the strict coincidence between the periodicity of the source flux variations and that of the dithering pattern, as shown in Fig. 4, top panels. More examples are shown in de la Vega et al. (2018).
- Again with a pattern matching the dithering motion, some very brief (≤10 sec) conspicuous drops in flux, by up to 1 mag or more, occur occasionally. These are mostly due to the source passing over a hot spot, and can be essentially culled by removing datapoints flagged as affected by hot spots. One of the advantages of the fast-cadence time-resolved photometry is that such very limited (in time) events can be excluded, and good datapoints over most of the observing time be used, as opposed to just one integrated measurement over the whole exposure that would include good and bad events (third-row right-panel in Fig. 4, NUV). In rare cases, a few extreme flux drops occur in a visit, not related to hot spots but possibly to misalignments between FUV and NUV images (de la Vega et al. 2018).
- One-time shifts in flux by up to a few tenths of a magnitude (“jumps”) occur occasionally during an observation, more often in FUV than in NUV.
- “sagging”: a sinusoidal-like variation with period equal to the observation duration, resembling a “sagging” or “heaving” of the source flux, occur at times. Such artifact, as well as the others above mentioned, does not occur in all the observations of the same source; it occurs when sources trace a long loop across the detector rather than a spiral dithering pattern during the visit (see de la Vega et al. 2018). We examined other field sources in the same observations: they are affected by the same artifact although not necessarily in the same direction, e.g., some may show heaving instead of sagging of the flux (Fig. 4, bottom panels). These facts again suggest, as is the case for the triangle-wave artifact variation, spatial inhomogeneities of the detector response on a scale smaller than they were characterized and accounted for by the calibration.^5^

The occasional but not infrequent occurrence of such conspicuous instrumental effects hampers any automatic search for variability within large samples, such as for example by looking for maximum magnitude difference within all datapoints of a light curve compared to the measurements errors. Most variations, except for flares (e.g., Million et al. 2017) will be smaller than the artifacts, and yet significant, and physically relevant. We refer to the comprehensive paper of de la Vega et al. (2018) for recipes and caveats about discerning artifacts occurrences.

Lastly, we show examples of real source variations on the shortest time scales probed at UV wavelengths to our knowledge. One example is the well-known dwarf nova Z Cam, thoroughly investigated at optical wavelengths. Figure 5 shows light curves for two (out of 10 total) GALEX observations, sampled with a time resolution of 5 seconds, displaying variability by up to 0.3 mag on time-scales of ∼hundreds of seconds; over the whole sample of GALEX observations of Z Cam, from 2003 to 2007, flux varies by up to ∼1 mag. The flux of this bright source falls in the non-linear response regime for GALEX, however some of the variations are more conspicuous than non-linearity effects (e.g., Fig. 2); as a test, we extracted light curves of the other three brightest stars included in the fields during the Z Cam observations^6^: these are stable within the uncertainties (de la Vega et al. 2018). Another, very different example is PG1144+005 (Fig. 4, second row—right panel), where possible short-term pulsations are however difficult to discren due to the dithering-induced artifact variations.

More information on GALEX data, science catalogs and projects can be found at the author’s $\mathit{UVSKY}$ web site http://dolomiti.pha.jhu.edu/uvsky.

## Footnotes

1 At “visit” level; a “visit” is a single observation with GALEX; the number of measurements from “coadds” is 214,499,551 and these still include some multiple measurements of the same source, see Bianchi et al. (2017).

2 64,551 with both FUV and NUV detectors exposed, 47,239 of which from the AIS survey.

3 Details on the asociation and identification of sources are given in Bianchi et al. (2018b).

4 In more detail, this parameter does not always provide a good restriction, but often an excessive restriction, especially for bright sources: for example, some of the CALSPEC calibration stars have higher ellipticity, anyway we show a reduced sample for clarity of the figure.

5 Such inomogeneities are in fact one of the very reasons for dithering during the exposure, along with avoiding hot spots and detector fatigue from bright sources.

6 Two have NUV≈13.6 ABmag and one has NUV≈14.3 ABmag.
